# Spatially Placed Arbiter PUFs on an FPGA for UAV Swarm Sensing Nodes: A Proof-of-Concept Evaluation

**DOI:** 10.3390/s26216719

**Published:** 2026-10-23

**Authors:** Spyridon Blionas, Antonios S. Andreatos

**Affiliations:** 1 Department of Informatics and Telecommunications, University of the Peloponnese, GR-22131 Tripoli, Greece; 2 Division of Computer Engineering and Information Science, Hellenic Air Force Academy, Dekeleia, GR-13671 Attica, Greece

**Keywords:** Physical Unclonable Functions, Arbiter PUF, spatial placement, FPGA, UAV swarm, Internet of Drones, reliability, inter-placement uniqueness, cross-device comparison, multi-APUF architecture

## Abstract

Spatially placed Arbiter Physical Unclonable Functions (APUFs) exploit FPGA placement diversity to generate placement-dependent challenge–response mappings while preserving an identical logical architecture. This work presents a proof-of-concept evaluation of twenty fine-grained spatial profiles of an eight-chain, eight-stage APUF. Each profile was implemented and measured separately using one full-device configuration, and all 256 challenges were evaluated with twenty repeated measurements per profile. On the first Xilinx VC709 board, the prototype achieved 99.8843% mean raw-bit reliability, 99.0820% mean 8-bit block reliability, 47.9858% majority-response uniformity, and 47.6514% mean inter-placement uniqueness. The same twenty constrained implementations were evaluated on a second VC709 board solely to investigate cross-device behavior. Corresponding profiles on the two boards differed by 2.0703% on average, whereas the 380 nonmatching cross-device profile pairs differed by 47.6537% on average. The largest corresponding-profile distance was 3.5645%, while the smallest nonmatching-profile distance was 35.4492%, yielding an empirical separation of 31.8847 percentage points. These results indicate that deterministic placement and routing define the dominant profile personality, while device-specific manufacturing variation contributes a smaller residual. The present work does not implement concurrent multi-APUF operation or an end-to-end authentication protocol. The measurements instead motivate near-term work in which twenty or more qualified APUFs are instantiated concurrently on one FPGA, selected instances provide wider stable internal response material, and a cryptographic key-derivation process supplies 128-bit keys for an Ascon-AEAD128-protected UAV authentication and sensor-data channel.

## 1. Introduction

Unmanned Aerial Vehicle (UAV) swarms are increasingly employed as distributed sensing platforms in environmental monitoring, infrastructure inspection, emergency response, remote surveillance, and other applications. In such systems, multiple UAV nodes collect, process, and exchange sensor information through wireless communication channels. The mobility of the nodes, the accessibility of the wireless channel, and the possibility of physical capture introduce risks including device impersonation, unauthorized node replacement, sensor-data modification, replay, and extraction of cryptographic credentials.

Physical Unclonable Functions (PUFs) provide an attractive hardware root of trust because they derive responses from physical characteristics rather than relying exclusively on permanently stored secret keys [1,2,3,4,5]. PUF-assisted authentication has already been considered for Internet of Drones (IoD) environments, including mutual authentication, key agreement, and resistance to physical capture [6,7,8,9,10,11]. The present study complements protocol-oriented approaches by experimentally investigating a reconfigurable Field Programmable Gate Array (FPGA) PUF primitive intended for UAV sensing nodes.

An Arbiter PUF (APUF) is a delay-based PUF in which two nominally equivalent signal paths are configured by a challenge vector. An arbiter determines which path transition arrives first and produces one response bit [2,3]. Because propagation delays depend on physical implementation characteristics, nominally equivalent circuits may produce distinct responses. However, a conventional APUF exposes a static challenge–response function that can be approximated by machine-learning models when sufficient challenge–response pairs are collected [12,13].

Although APUFs have been extensively investigated for lightweight authentication and key establishment, most existing FPGA implementations assume a fixed physical realization after device programming. Consequently, a conventional FPGA APUF exposes a single static challenge–response function throughout its operational lifetime. Once a sufficiently large set of challenge–response pairs has been collected, this fixed mapping may become susceptible to modeling attacks, depending on the PUF architecture, the available challenge space, and the attack methodology. Numerous countermeasures have therefore been proposed to improve robustness and resistance against modeling attacks, including exclusive-OR (XOR) APUFs, feed-forward structures, controlled PUFs, reconfigurable PUF architectures, and protocol-level protections [1,4,6,7,8,9,10,11]. However, many of these approaches either increase hardware complexity, incur additional implementation overhead, or modify the logical PUF architecture itself.

The inherent reconfigurability of modern FPGAs provides an alternative opportunity that has received comparatively limited experimental investigation. Instead of changing the logical structure of the PUF, the physical implementation itself can be modified by relocating an identical design to different regions of the FPGA fabric. Because signal propagation delays are strongly influenced by the selected configurable logic blocks, routing resources, switch matrices, and placement decisions, identical logic implementations may exhibit measurably different delay characteristics and therefore different challenge–response mappings. While previous work has investigated reconfigurable FPGA PUFs based on programmable delay elements, secure reconfiguration techniques, and configurable switch-block architectures [4,14,15,16,17], the systematic characterization of multiple independently constrained placement profiles of an otherwise unchanged FPGA APUF implementation remains comparatively unexplored.

Among FPGA-based PUF implementations, APUFs are particularly interesting because their responses are directly influenced by physical delay characteristics, which are known to depend on placement and routing decisions. Previous studies have shown that physical implementation can substantially affect reliability, bias, and uniqueness [18,19,20,21]. Understanding how fine-grained placement influences response behavior is important both for FPGA PUF evaluation and for prospective deployment scenarios in which multiple spatial profiles may be used for device authentication, profile selection, or reconfigurable security architectures.

This observation motivates the use of spatially placed Arbiter PUF profiles. Rather than constructing multiple different PUF architectures, the present study preserves an identical logical APUF implementation while deliberately relocating the design among multiple FPGA locations. Each separately generated spatial profile uses different physical logic and routing resources and therefore realizes a placement-dependent challenge–response mapping. The current proof of concept evaluates these profiles one at a time. A future architecture may instantiate a qualified set of such APUFs concurrently on one FPGA and select one or more instances during authentication.

The concept is relevant to future UAV swarm sensing systems because concurrently instantiated, spatially separated APUFs could provide a selectable hardware-bound source without requiring a large library of full-device bitstreams. However, the present measurements do not demonstrate concurrent operation, runtime instance selection, response combination, cryptographic key reconstruction, or an operational UAV authentication protocol. They evaluate the underlying placement-defined reliability and diversity needed before such integration can be attempted.

To investigate this placement-dependent behavior, this paper presents a proof-of-concept implementation of an eight-chain, eight-stage Spatially Reconfigurable Arbiter PUF (SR-APUF) implemented on a Xilinx Virtex-7 FPGA. Twenty independently constrained spatial implementations were generated from a common logical design and experimentally evaluated using all 256 possible challenges under repeated measurements. Reliability, majority-response uniformity, inter-placement uniqueness, response diversity, parity consistency, and placement-dependent bias were analyzed to determine whether spatial relocation constitutes a practical mechanism for generating multiple stable PUF personalities on a single reconfigurable device.

It should be emphasized that the present implementation is an experimental characterization platform rather than a production-ready strong PUF. The objective of this work is to systematically investigate the influence of fine-grained FPGA placement on APUF reliability, uniformity, and inter-placement diversity. Consequently, the study does not claim deployment-ready authentication, cryptographic key generation, machine-learning resistance, or operational UAV security validation. Instead, it focuses on providing the experimental evidence needed to assess the feasibility of placement-defined APUF profiles and to motivate future implementations.

A second physical VC709 was subsequently evaluated using the same twenty fine-grained profiles and acquisition procedure. This permits two complementary comparisons: inter-placement diversity within one device and corresponding-profile behavior across two devices. The new measurements reveal a hierarchical structure in which the profile determines the dominant response personality, while the physical die contributes a smaller device-specific perturbation.

The paper concludes by outlining a prospective integration path. In the near-term architecture, twenty or more independently constrained APUFs would coexist in a single FPGA configuration, a keyed controller would activate one or more qualified instances, and stable internal response material would be processed by helper-data mechanisms and a cryptographic key-derivation function. Integration with Ascon-AEAD128 (authenticated encryption with associated data) is retained as future work and is not implemented or evaluated in the present study.

The remainder of this paper is organized as follows. Section 2 presents the background, related work, evaluation metrics, and the prospective multi-APUF architecture. Section 3 describes the spatially placed APUF architecture, the placement profiles, and the parity encoder. Section 4 describes the FPGA implementation, the arbiter and measurement behavior, and the host interface. Section 5 presents the experimental methodology and measurement conditions. Section 6 presents the experimental results, including reliability, uniformity, uniqueness, parity consistency, and cross-device observations. Section 7 discusses placement-related limitations and potential implementation improvements. Section 8 discusses the application to UAV swarm sensing nodes. Section 9 discusses security implications, limitations, and directions for future work. Section 10 summarizes the conclusions.

## 2. Background and Related Work

### 2.1. Arbiter Physical Unclonable Functions

A conventional APUF contains a sequence of challenge-controlled switching stages. Each stage implements a 2 × 2 switch with two inputs and two outputs. Depending on challenge bit c_i_, the two paths pass through the stage directly or cross.

*c_i_* = *0*: *a_out_* = *a_in_*, *b_out_* = *b_in_* (1)

*c_i_* = *1*: *a_out_* = *b_in_*, *b_out_* = *a_in_* (2)

A transition is launched simultaneously into the two paths. After passing through all stages, the two signals enter an arbiter. The arbiter produces a binary response according to which transition arrives first [2,3]. In an ideal implementation, the two nominal paths are balanced and the observed delay difference is dominated by uncontrollable physical variation. On an FPGA, deterministic routing differences can exceed the smaller manufacturing-dependent delay differences, making placement and routing control essential [14,15].

### 2.2. FPGA APUF Implementation

FPGA implementations of APUFs use lookup tables (LUTs) to realize challenge-controlled multiplexers and LUT-based, latch-based, or flip-flop-based structures to implement the arbiter [4,14,15]. FPGA configurability enables controlled placement experiments, but synthesis and implementation tools may alter hierarchy, map logic to different LUT inputs, or select asymmetric routes [14,15,22,23,24]. Reconfigurable APUFs have also been investigated using configurable 4 × 4 switch blocks and Series-7 FPGA implementations [16,17]. The proposed SR-APUF focuses on spatial relocation of an unchanged 2 × 2-switch APUF architecture; the physical profile, rather than a changed logical PUF primitive, defines the active personality.

### 2.3. Evaluation Metrics

#### 2.3.1. Uniformity

Uniformity is the percentage of response bits equal to one and is a standard PUF quality metric [4,5]. For Q challenges, m response bits, and spatial profile i:

(3) $${\mathrm{Uniformity}}_{i}=\frac{\sum_{k=1}^{Q}\sum_{b=1}^{m}R_{i,k,b}}{\mathrm{Qm}}\times 100\%$$

where i denotes the spatial profile index, k denotes the challenge index, b denotes the response-bit index, Q is the total number of evaluated challenges, m is the number of response bits, and *R_i_*_,*k*,*b*_ ∈ {0,1} is the value of response bit b produced by profile i for challenge k.

The ideal value is 50%.

#### 2.3.2. Reliability

Reliability measures whether one implementation reproduces the same response over repeated evaluations and is commonly evaluated under repeated and environmental measurements [4,5,15]. A majority-vote response is used as the reference. For T repeated measurements,

(4) $${\mathrm{BER}}_{i,\;k}=\frac{\sum_{t=1}^{T}\mathrm{HD}\left(R_{i,k}^{\left(t\right)}\;,R_{i,\;k}^{\left(\mathrm{ref}\right)}\right)\;}{\mathrm{Tm}}$$

where T is the number of repeated measurements; m is the response width; HD(⋅,⋅) denotes the Hamming distance; $R_{i,k}^{\left(t\right)}$ is the t-th measurement; $R_{i,k}^{\left(\mathrm{ref}\right)}$ is the majority-vote reference response.

*Reliability_i_*_,*k*_ = (1 − *BER_i_*_,*k*_) × 100% (5)

where *BER_i_*_,*k*_ is the normalized bit-error rate defined above.

#### 2.3.3. Inter-Placement Uniqueness

Traditional PUF uniqueness compares different physical chips [4,5]. The present prototype compares different placements on one chip; the appropriate term is inter-placement uniqueness or spatial-configuration diversity. For M profiles and Q challenges,

(6) $$U_{\mathrm{place}}=\frac{2}{M\left(M-1\right)Q}\sum_{i<j}\sum_{k=1}^{Q}\frac{\mathrm{HD}\left(R_{i,k},\;R_{j,k}\right)}{m}\times 100\%$$

where M denotes the number of spatial profiles; Q denotes the number of evaluated challenges; m denotes the response width; *R_i_*_,*k*_ and *R_j_*_,*k*_ are the responses of profiles i and j to challenge k; *HD*(*R_i_*_,*k*_, *R_j_*_,*k*_) denotes the Hamming distance.

The ideal value for independent and balanced profiles is approximately 50%.

### 2.4. FPGA APUF Uniqueness, Evaluation Scale, and Routing Imbalance

Large-scale FPGA studies have shown that APUFs may exhibit excellent intra-device repeatability while their inter-device uniqueness is reduced by deterministic placement and routing imbalance. Hori et al. evaluated 45 Virtex-5 devices and emphasized both rigorous definitions of randomness, steadiness, correctness, diffuseness, and uniqueness and the importance of confidence intervals across physical devices [18]. Machida et al. reported conventional FPGA APUF uniqueness below 5% in one experiment and proposed the Double Arbiter PUF mode of operation, which raised three-chain inter-device uniqueness to approximately 50% but reduced steadiness substantially [19]. Akhil and Supriya compared horizontal, vertical, and zigzag placement patterns and reported that physical separation and geometric similarity influence FPGA APUF uniqueness [20]. He et al. introduced a bit-self-test mechanism that identifies reliable response bits from measured delay deviation and reported improved response quality after screening [21]. These findings are directly relevant to interpreting the present results: placement geometry, path balance, and response selection can improve specific quality metrics, but may also change stability and the physical meaning of the retained responses.

Deep-learning studies have additionally modeled conventional APUF, XOR-APUF, feed-forward APUF, and Multi-APUF structures implemented on FPGA with prediction accuracy no lower than 93.56% in the reported evaluation [25]. Spatial rotation is therefore not treated as proof of machine-learning resistance. The intended security benefit is to restrict clean challenge–response pair (CRP) collection, shorten the useful lifetime of a profile-specific model, and enable revocation, while cryptographic authentication protects the protocol transcript.

### 2.5. Prospective Multi-APUF Authentication Architecture

Conventional PUF authentication can be expressed as R = f_D_(C), where D denotes the physical device and C the applied challenge and R is the response. The present work experimentally characterizes placement-dependent mappings R = f_D_(P,C), where P identifies a distinct spatial implementation profile within the FPGA fabric. Although the experimental validation focuses on individual APUF instances, the proposed architecture can be extended to support concurrent deployment of multiple qualified APUFs on the same FPGA.

In the envisioned implementation, a set of APUFs is instantiated simultaneously and controlled through a runtime selection vector received from the verifier. The selection vector determines which APUF instances are activated for a given authentication session, while non-selected instances remain idle to minimize switching activity and dynamic power consumption. The selected APUFs evaluate the common challenge in parallel, and their responses are collected through a lightweight response-packing mechanism that sequentially gathers the outputs of the selected instances and forms the authentication response transmitted to the verifier.

This architecture enables runtime profile selection and supports authentication schemes based on different subsets of spatially distributed APUFs. Consequently, the authentication function can be generalized as R = f_D_(S,C), where S denotes the verifier-selected subset of qualified APUF instances.

Nevertheless, this architecture has not been experimentally implemented or validated in the present study and should therefore be regarded as a prospective extension motivated by the observed placement-dependent behavior. Its practical deployment requires additional investigation, including the effects of concurrent APUF operation on routing resources, response correlation among colocated instances, power consumption, environmental reliability, communication overhead, and the performance of any associated key-reconstruction or authentication protocols.

## 3. Spatially Placed APUF Proof of Concept

### 3.1. Logical Architecture

The proof-of-concept architecture contains eight parallel APUF chains. Each chain contains eight switching stages controlled by the same 8-bit challenge. Each chain generates one response bit; the eight parallel chains therefore generate an 8-bit raw response.

**Figure 1:**
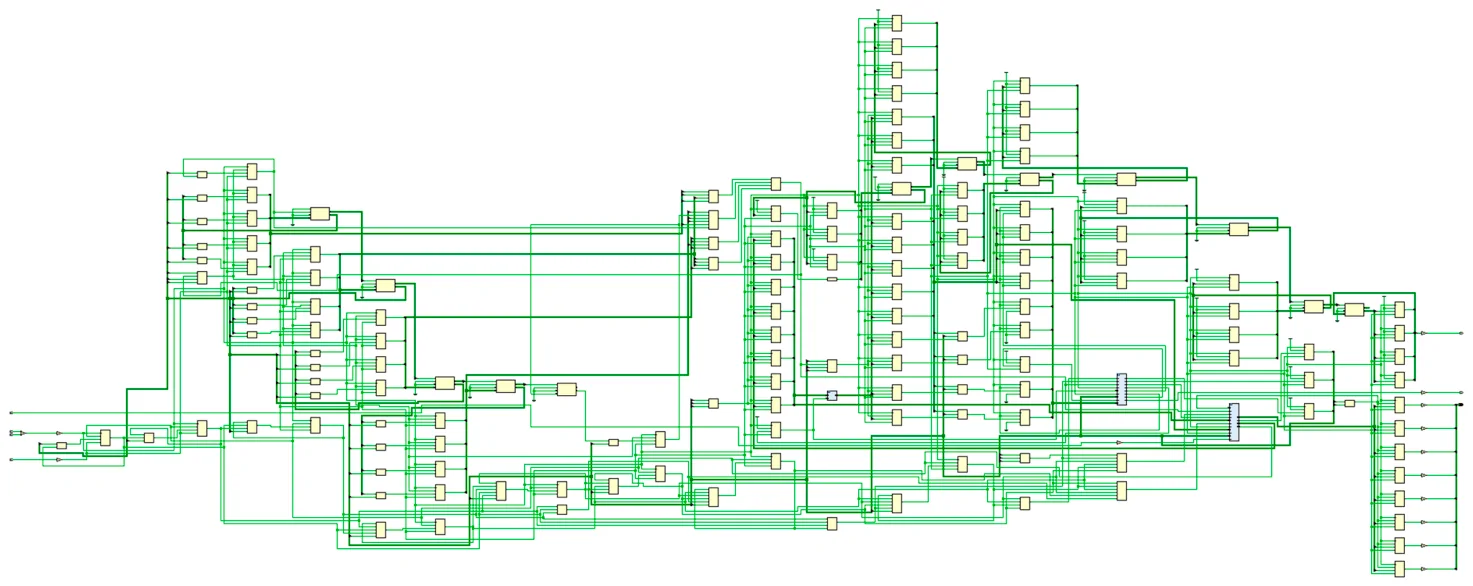
Eightfold APUF architecture. The design contains eight parallel Arbiter Physical Unclonable Function (APUF) chains, each comprising eight challenge-controlled switching stages. The yellow blocks represent 2 × 2 switching elements implemented using LUT-based multiplexers, while the green lines represent the two competing delay paths that propagate a common launch transition through the network. Depending on the value of the corresponding challenge bit, each switching stage either preserves or exchanges the upper and lower signal paths. At the end of each chain, an arbiter determines which path arrives first and generates one response bit. The eight response bits are combined to form the 8-bit response word.

In Figure 1, the green interconnections represent the two racing signal paths that carry the launched transition through the APUF. The yellow switching elements correspond to challenge-controlled 2 × 2 switch stages implemented using LUT-based multiplexers. Each challenge bit determines whether a stage preserves or exchanges the two signal paths before they propagate to the next stage. The arbiter at the end of each chain generates a single response bit according to the first arriving transition.

Each switching stage contains two 2:1 multiplexer functions. The complete prototype therefore contains 8 chains x 8 stages x 2 multiplexers = 128 multiplexer functions. Post-implementation utilization indicates that one chain uses sixteen three-input lookup table (LUT3) primitives for its eight switching stages, consistent with two separate multiplexer LUTs per stage.

### 3.2. Spatial Profiles

Twenty implementation profiles were constructed from one synthesized netlist of the design described in VHDL (VHSIC hardware description language). For every profile, an implementation-only Xilinx Design Constraints (XDC) file explicitly fixed the physical location (LOC) and the basic element of logic (BEL) site A6LUT of all 128 switch-stage LUT3 cells (8 chains × 8 stages × 2 multiplexers), and DONT_TOUCH directives preserved the intended structure during implementation. Post-implementation reports confirmed 128/128 multiplexer (MUX) LUTs with fixed LOC and BEL assignments for each verified profile (Section 5.1).

The constraints were applied only to the placement of these LUT cells. Routing resources, path symmetry between the two competing signal paths of each stage, and delay balancing across stages were not constrained and were selected automatically by Vivado (AMD Vivado Design Suite, v2021.1, AMD, San Jose, CA, USA). The measured differences between profiles therefore reflect the combined effects of the prescribed LUT placement and the Vivado-generated routing, rather than placement alone. Section 7.1 discusses the remaining implementation degrees of freedom and a more tightly controlled placement method.

### 3.3. Hamming(12,8) Parity Bits

The eight raw response bits are supplied to a Hamming(12,8) parity encoder, which forms a 12-bit codeword using the systematic arrangement P1, P2, D0, P4, D1, D2, D3, P8, D4, D5, D6, D7. The parity equations are:

*P1* = *D0 XOR D1 XOR D3 XOR D4 XOR D6* (7)

*P2* = *D0 XOR D2 XOR D3 XOR D5 XOR D6* (8)

*P4* = *D1 XOR D2 XOR D3 XOR D7* (9)

*P8* = *D4 XOR D5 XOR D6 XOR D7* (10)

The four generated values are used strictly as auxiliary codeword-integrity bits. They are deterministic functions of the raw response and do not add independent PUF entropy. The parity bits do not perform error correction, noise mitigation, fuzzy extraction, helper-data generation, or key reconstruction: no decoder or correction logic is implemented, and the raw response bits are never modified on the basis of the parity bits. The Hamming(12,8) parity encoder is included on the FPGA, and the parity relations are checked offline on the host (Section 6.6). Parity-invalid records observed in the measurements cannot presently be attributed to intrinsic PUF instability; they may equally arise from capture, synchronization, serialization, or communication effects.

## 4. FPGA Implementation

### 4.1. Hardware Platform

The SR-APUF was implemented on a Xilinx VC709 evaluation board containing an XC7VX690T-2FFG1761 Virtex-7 FPGA [26] (see Table 1). The device was selected as a proof-of-concept platform because its large configurable fabric supports numerous physically separated APUF placements. The relevant LUT, SLICEL (logic slice), SLICEM (logic-and-memory slice), and configurable logic block (CLB) organization is documented in the 7-series CLB guide [22], while the implementation and project flows follow the Vivado guidance in [23,24].

### 4.2. Switch-Stage Implementation

Each switch block is described behaviorally as two complementary multiplexer functions. In the routed implementation, Vivado maps the eight stages of one chain to sixteen LUT3 primitives, consistent with the 7-series LUT and slice architecture [22]. The utilization report of the placed design is summarized in Table 2, which lists the primitives. The 317 LUT primitives (LUT1 to LUT6) are packed into 269 physical slice LUTs (0.06% of the device), of which 128 are the fixed switch-stage LUT3 cells. The design further uses 157 registers (156 FDRE and one FDSE), 192 slices, 14 bonded I/O pads, and one global clock buffer. Each chain additionally contains the two LUT2 primitives of its NAND-latch arbiter and no flip-flop.

**Figure 2:**
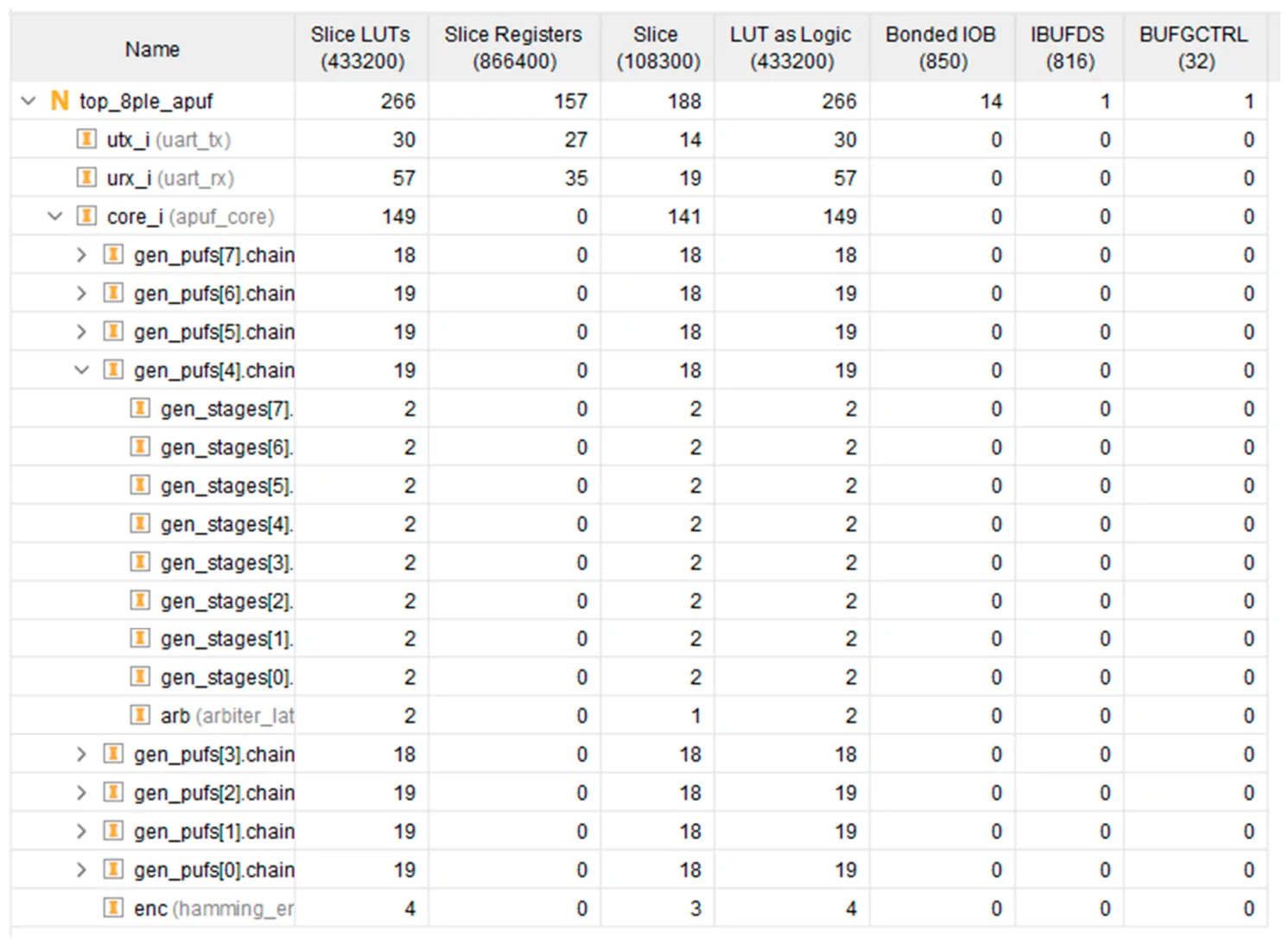
Top-level Vivado resource-utilization summary of the implemented prototype. The figure shows the hierarchy and resource consumption reported after implementation of the complete FPGA design. The identifier core__i_ denotes the main SR-APUF processing block, while utx__i_ and urx__i_ correspond to the universal asynchronous receiver–transmitter (UART) transmitter and receiver interfaces used for challenge delivery and response acquisition. The reported values indicate the number of LUTs, registers, slices, and associated FPGA resources used by each module and highlight the small resource footprint of the proof-of-concept implementation relative to the XC7VX690T device capacity. The number in parentheses under each column title is the total amount of that resource available on the XC7VX690T device. The figure shows one implemented profile, which differs from the profile of Table 2, so the totals differ slightly.

Figure 2 is included primarily to demonstrate the resource requirements of the prototype. The hierarchy labels correspond to Vivado-generated module instances. The core_i module contains the APUF implementation and associated control logic, whereas the UART modules provide communication with the host computer. The utilization report confirms that the prototype occupies only a small fraction of the available FPGA resources, leaving substantial capacity for additional APUF instances or future authentication logic.

### 4.3. Arbiter and Measurement Behavior

The control logic uses the 200 MHz system clock, but the APUF delay paths are not driven by a free-running clock. For each received UART challenge, the controller first holds the common launch input low and applies the challenge vector to the switching network. After allowing the challenge-controlled MUX configuration to stabilize for 1 µs (200 clock cycles), a single rising transition is launched simultaneously into both competing delay paths.

Propagation through the APUF and race resolution within the arbiter occur on the nanosecond scale. The arbiter of each chain is a cross-coupled NAND set–reset latch (VHDL entity arbiter_latch: q = NOT(top AND qn), qn = NOT(bot AND q), response = q), implemented with two LUT2 primitives that form a combinational loop. The loop is preserved by DONT_TOUCH and ALLOW_COMBINATORIAL_LOOPS attributes and is excluded from static timing analysis by a false-path constraint. While both inputs are low, the latch rests in its idle state (q = qn = 1), which is the precharge state of the evaluation. After the launch edge, the path whose transition arrives first at the latch determines the final state: the response is 0 when the top path arrives first and 1 when the bottom path arrives first. No reset gates are inserted into the race paths or the latch, and no LOC or BEL constraint is applied to the arbiter LUTs, whose placement and input routing are selected by Vivado (Section 5.1). After the 1 µs challenge-settling interval, the launch input performs a single 0 → 1 transition and remains high for 200,000 clock cycles (1 ms). This 1 ms interval is not required for propagation or arbitration; it is a fixed delay that guarantees a stable arbiter state before sampling and provides deterministic response acquisition. After this interval, the controller captures the complete 12-bit codeword (raw response and Hamming parity bits) in a snapshot register, the two UART response bytes are transmitted from this snapshot, and the launch input then returns to logic 0, which restores both paths to their idle (precharged) state before the next evaluation.

Each measurement therefore corresponds to a single launch event followed by a single response capture. No free-running oscillation or repeated evaluation occurs during the measurement process.

Static timing analysis does not cover the race paths: the arbiter nets are excluded by a false-path constraint, and no delay or skew constraints were applied to the competing paths. The only timing constraint is the 5 ns (200 MHz) system-clock constraint, which applies to the control logic. Per-path post-route delay and skew values were not extracted in this study. Consequently, no representative post-route path-delay figures are reported, and the delay asymmetry of the competing paths is characterized only indirectly, through the measured responses (Section 6). Post-route timing analysis of the competing paths is identified as a recommended extension of this work.

### 4.4. Host Interface

The host computer sends one challenge byte through a 115,200-baud UART interface. Following the fixed challenge-settling and race-wait intervals described in Section 4.3, the FPGA returns two bytes containing the twelve-bit codeword (captured as a single snapshot). On-board light-emitting diodes (LEDs) display the challenge during the settling state and the response during transmission.

## 5. Experimental Methodology

### 5.1. Spatial Configurations

Twenty bitstreams were generated from one synthesized VHDL netlist using twenty autonomous implementation-only XDC files. Each XDC fixes the LOC and A6LUT BEL of all 128 switch-stage LUT3 cells. Post-implementation reports confirmed 128/128 MUX LUTs with fixed LOC and BEL assignments for each verified profile.

**Figure 3:**
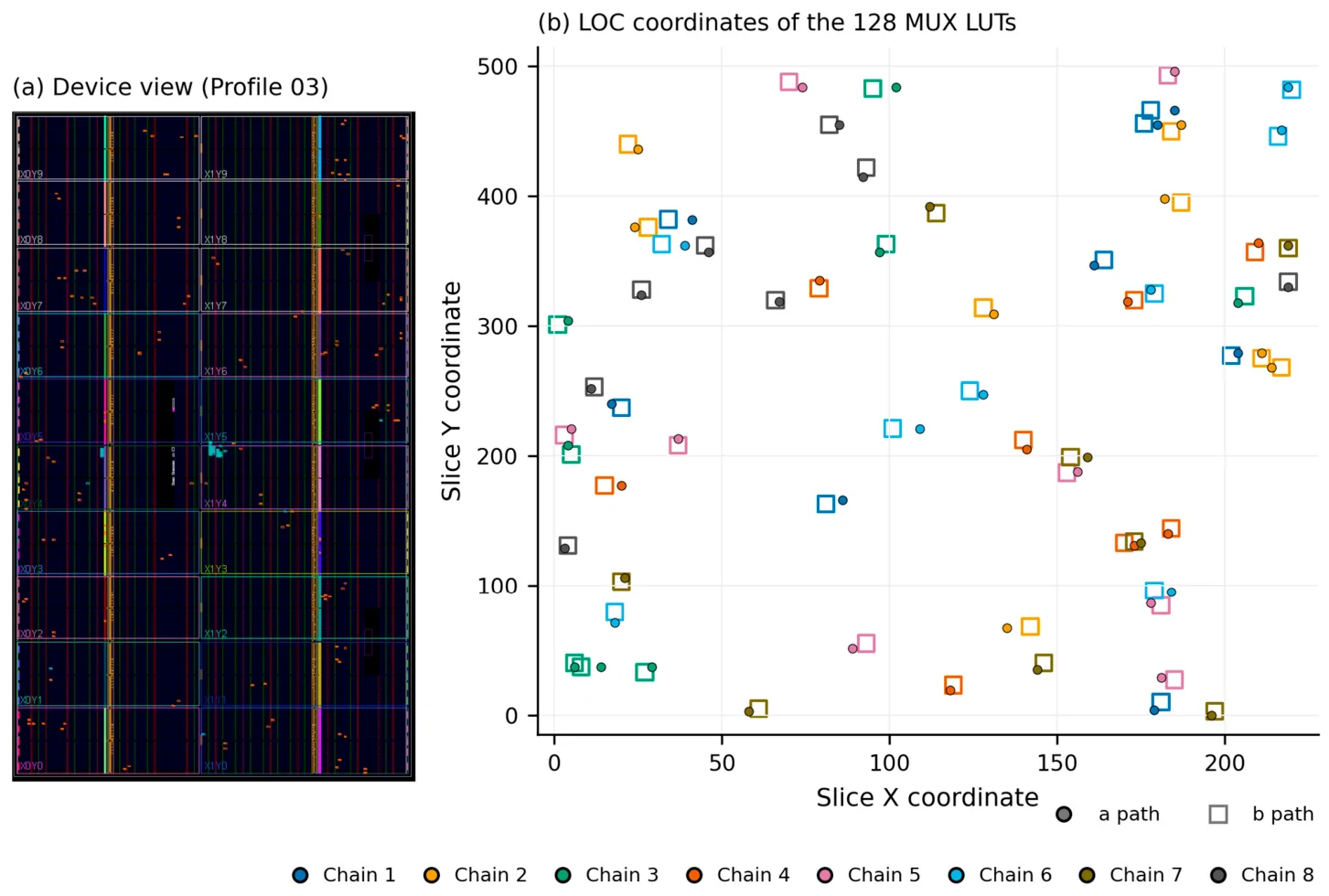
FPGA floorplan and chain-resolved MUX placement for spatial Profile 03. (**a**) Vivado Device View of the implemented XC7VX690T design. (**b**) Chain-resolved coordinate plot showing the exact slice locations of the 128 LUT3 switch-stage MUX cells extracted from the post-implementation placement report. Each color identifies one of the eight parallel APUF chains, filled circles denote a-path LUTs, and open squares denote b-path LUTs. Every chain contains eight challenge-controlled switching stages implemented using sixteen LUT3 MUX elements, corresponding to separate a-path and b-path signal routes. The coordinate plot therefore visualizes the complete physical placement of all switch-stage MUX resources associated with the eight-chain, eight-stage APUF implementation.

Figure 3 illustrates the physical realization of representative spatial Profile 03. Equivalent placement reports were generated and verified for all twenty spatial profiles.

**Figure 4:**
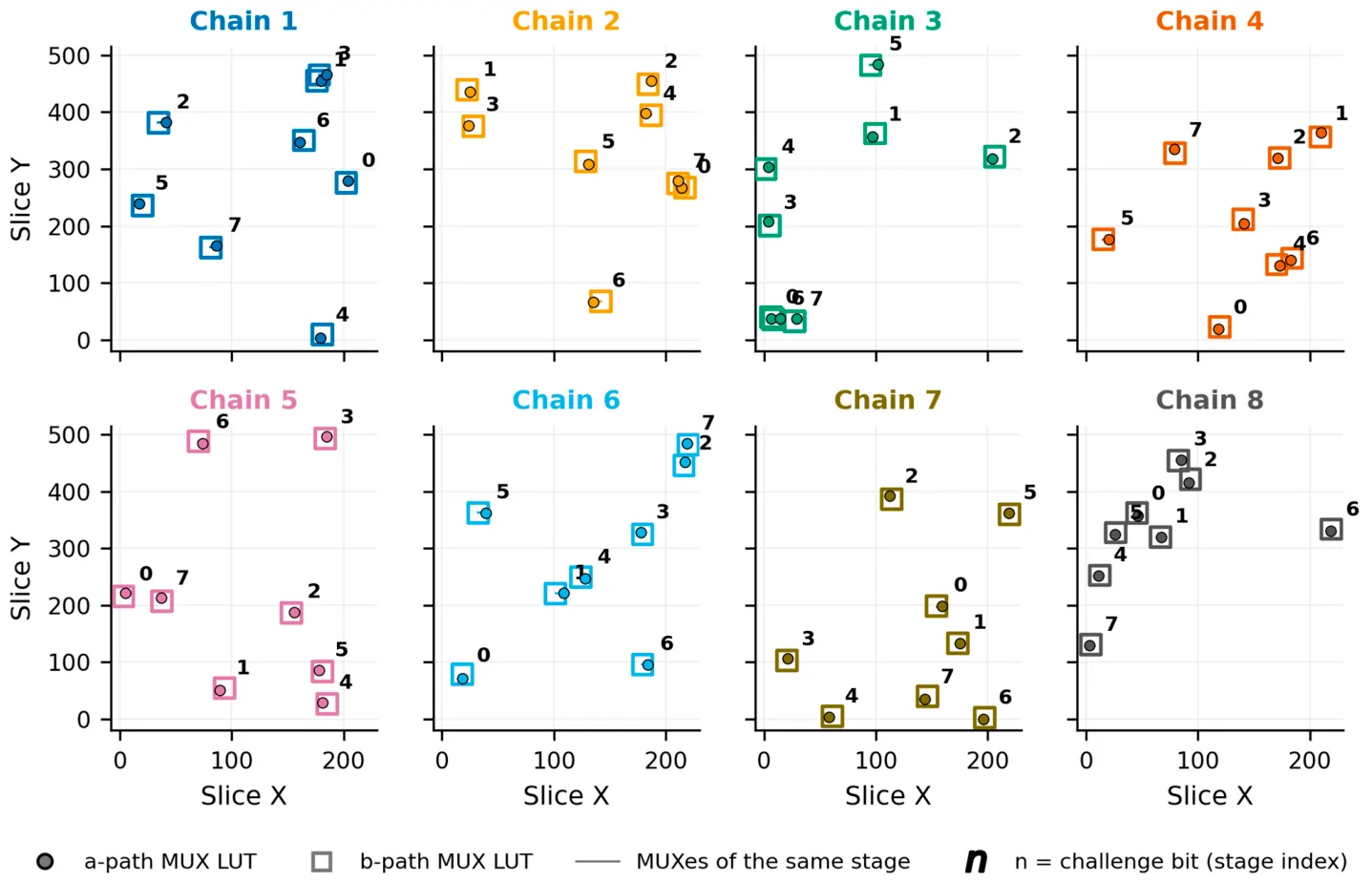
Detailed physical placement of the eight APUF chains in spatial Profile 03. Each panel represents one parallel APUF chain. Circular and square markers identify the upper (a-path) and lower (b-path) MUX LUT locations, respectively. Labels 0 through 7 identify the challenge-controlled switching stages, and connecting lines join the two MUXes belonging to the same stage. The figure is intended to illustrate the overall spatial distribution of the chain elements and switching stages. Exact slice coordinates, chain-resolved placement locations, and detailed placement information are provided in Figure 3 and the corresponding placement reports.

Post-implementation placement reports were generated for every spatial profile using friendly logical identifiers of the form MUX_c,s,p_, where c identifies the PUF chain, s identifies the controlling challenge bit, and p identifies the a or b signal path. Figure 3 and Figure 4 provide the complete chain- and stage-resolved placement visualization for representative Profile 03. The corresponding report contains 128 MUX records and confirms fixed LOC and BEL assignments for every MUX LUT. Equivalent machine-readable reports were generated for the remaining spatial profiles and are provided in the Supplementary Materials together with the bitstreams of all profiles.

DONT_TOUCH directives were applied to preserve the intended structure during implementation. Post-implementation verification confirmed successful realization of all specified placements. Routing resources, path symmetry between the competing signal paths, and delay balancing were not constrained; routing was selected automatically by Vivado. Consequently, the measured differences between profiles reflect the combined effects of the prescribed LUT placement and the Vivado-generated routing, rather than placement alone.

Each profile is defined by a standalone XDC file that contains 128 LOC and 128 BEL constraints, one pair for every switch-stage LUT3 cell, selected by exact cell-name filters. The 128 locations are distinct sites, and the placements were generated off-line from a recorded pseudo-random seed. In Profile 03, the two LUTs of the same stage are placed within a Manhattan distance of 2 to 8 sites of each other. Besides the pin assignments, the XDC defines the 5 ns system-clock constraint, enables combinational loops on the arbiter nets, and declares false paths through them. No timing, delay, or routing constraint is applied to the race paths.

### 5.2. Challenge and Response Acquisition

For every profile, the bitstream was loaded onto the FPGA, the host transmitted the selected 8-bit challenge or challenge sequence, and the FPGA returned the 8-bit raw response and four parity bits. Each measurement was repeated twenty times. Raw response bits, rather than parity-extended codewords, were retained for PUF uniqueness calculations.

All 256 possible 8-bit challenges were evaluated. The complete dataset contains 20 profiles × 256 challenges × 20 repetitions = 102,400 acquisitions. The raw capture files of the first board are provided in the Supplementary Materials.

### 5.3. Reference Response and Reliability

For each profile and challenge, the reference response is calculated through per-bit majority voting over the twenty measurements. The normalized Hamming distance between each measured response and the corresponding majority-vote reference is interpreted as the bit-error rate (BER). Reliability is then calculated as:

Reliability = (1 − BER) × 100% (Equation (5))

Consequently, the reliability values reported in Section 6 directly reflect the measured Hamming-distance statistics relative to the majority-vote reference responses.

### 5.4. Inter-Placement Uniqueness

Twenty profiles produce 190 pairwise comparisons. For every pair of profiles (i < j), the majority-vote responses of all 256 challenges (8 bits each, i.e., 2048 bits per profile) are compared bit by bit, and the Hamming distance between the two 2048-bit signatures is normalized to 2048 bits and expressed as a percentage, which is equivalent to the average normalized Hamming distance of the 8-bit responses over the 256 challenges, as defined in Section 2.3.3, a value of 50% indicates that the two placements produce statistically unrelated response signatures, whereas values close to 0% would indicate placements that are practically indistinguishable; the ideal value is therefore 50%. The mean, median, standard deviation, minimum, and maximum over the 190 pairs and the complete pairwise matrix are reported in Section 6.2. The uniqueness values of all 190 profile pairs are provided in the Supplementary Materials.

### 5.5. Uniformity and Per-Bit Bias

Uniformity is calculated independently for each profile. Per-bit bias is also evaluated to identify response positions that remain predominantly zero or one across challenges and profiles. These measurements help determine whether systematic routing bias limits inter-placement uniqueness.

### 5.6. Operating Conditions

Ambient temperature was 28 degrees Celsius, supply-voltage of VC709 was 12 Volts, power-cycle procedure was not necessary, there was no background FPGA activity, and the twenty repeats were collected after reconfiguration/reset of the FPGA.

The reported reliability values were obtained from repeated measurements performed under the nominal operating conditions described above. Consequently, the reported mean raw-bit reliability should be interpreted as a measure of repeatability under the tested laboratory conditions rather than a complete characterization of operational PUF reliability. The present study did not evaluate the effects of temperature variation, supply-voltage perturbation, long-term aging, or concurrent FPGA activity. In addition, reference responses and reliability values were derived from repeated measurements collected under the same operating conditions rather than from independent enrollment and evaluation sessions. Accordingly, the reported reliability results should be regarded as baseline laboratory repeatability measurements. Comprehensive environmental characterization and deployment-level reliability assessment remain important directions for future work.

### 5.7. Second FPGA and Cross-Device Comparison

The same twenty implementation profiles were evaluated on a second physical VC709 board. The two capture directories used identical filenames, profile_00_one_shot.txt through profile_19_one_shot.txt. Each file contained twenty complete sweeps of all 256 challenges. For every device and profile, a 2048-bit majority signature was formed from 256 challenges multiplied by eight raw response bits. The four parity bits were excluded from uniqueness calculations.

Corresponding-profile cross-device distance compares profile p on the first board with profile p on the second board. A complete 20 × 20 matrix additionally compares every profile on the first board with every profile on the second. Secure Hash Algorithm (SHA-256) hashes were retained to detect accidental duplicate captures. The capture files of both boards are provided in the Supplementary Materials. The twenty profiles characterize variation across spatial implementations for one physical board pair; they do not constitute twenty independent device pairs.

## 6. Experimental Results

Dataset validation confirmed 20 distinct capture hashes and 20 distinct majority signatures. All 102,400 acquisitions passed structural validation; no duplicate profile datasets were found.

### 6.1. Reliability

**Figure 5:**
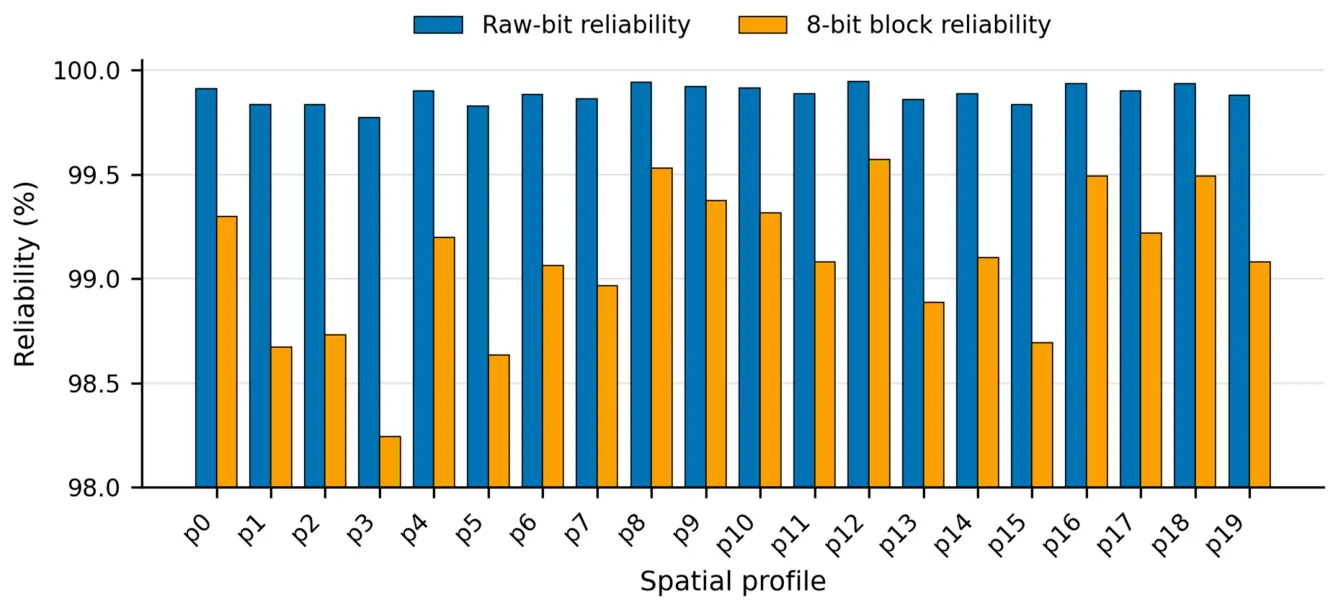
Raw-bit and 8-bit block reliability by spatial profile. The y-axis is truncated at 98%.

Across the twenty spatial profiles, mean raw-bit reliability, calculated using Equation (5), was 99.8843% (range 99.7729% to 99.9463%; population standard deviation (SD) 0.0446%). Mean 8-bit block reliability was 99.0820% (range 98.2422% to 99.5703%; population SD 0.3464%). The distinction is operationally relevant because a block is considered incorrect when any of its eight raw response bits differs from the per-challenge majority reference (Table 3 and Figure 5).

Reliability values approaching 100% indicate highly reproducible responses. Therefore, the measured mean raw-bit reliability of 99.8843% corresponds to a low disagreement rate relative to the majority-vote reference response and demonstrates strong repeatability under the evaluated laboratory conditions.

### 6.2. Inter-Placement Uniqueness

**Figure 6:**
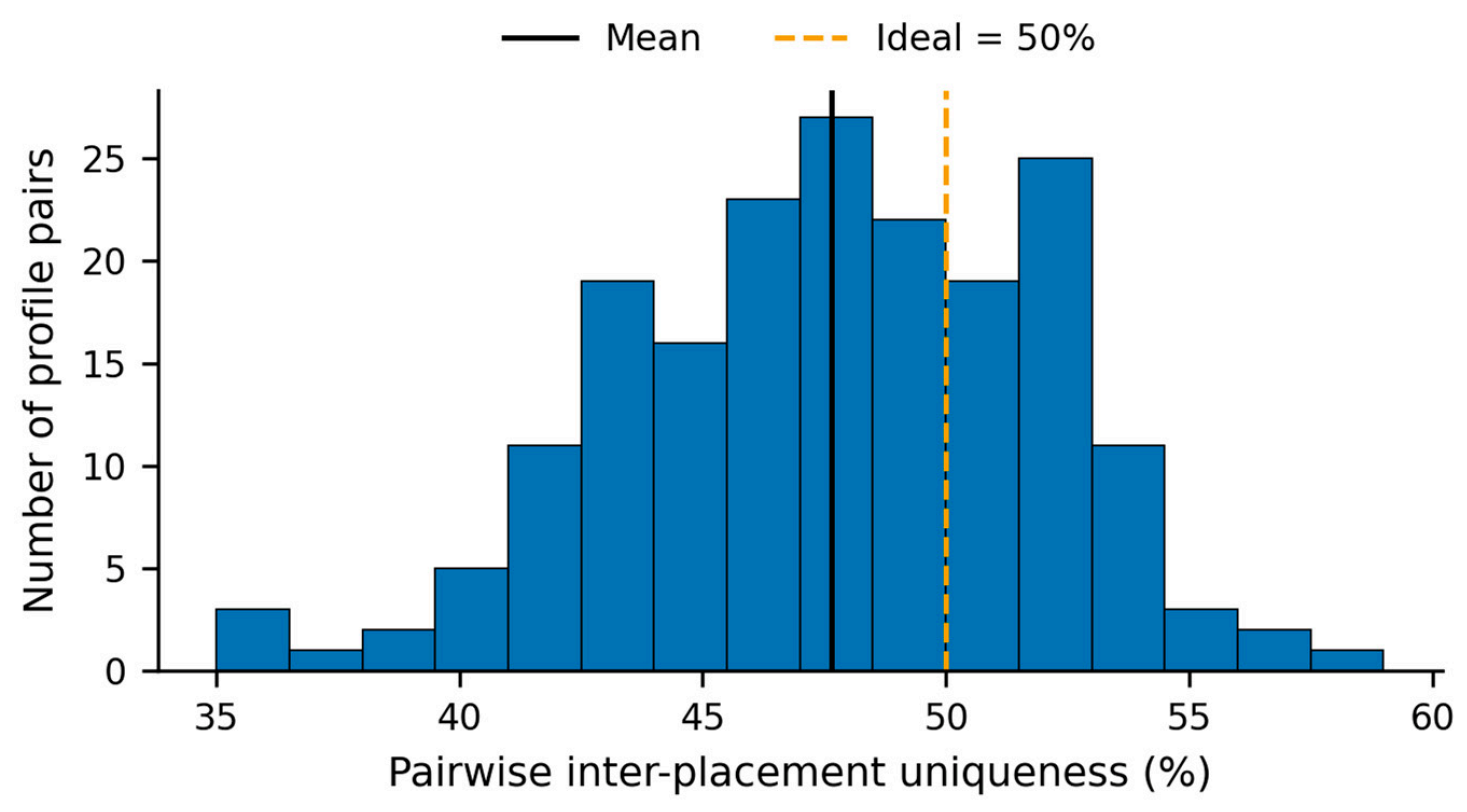
Distribution of the 190 pairwise uniqueness measurements. The solid line marks the mean and the dashed line the ideal value of 50%.

The twenty spatial configurations produced a mean inter-placement uniqueness of 47.6514%, calculated using Equation (6), across 190 profile pairs (Table 4 and Figure 6). The median was 47.8760%, the population standard deviation was 4.3261%, and the observed range was 35.8887% to 58.3496%. The mean is 2.3486 percentage points below the 50% ideal. No duplicate capture hashes or duplicate majority signatures were found; profile 02 versus profile 03 differs in 738 of 2048 majority-response bits (36.0352%).

**Figure 7:**
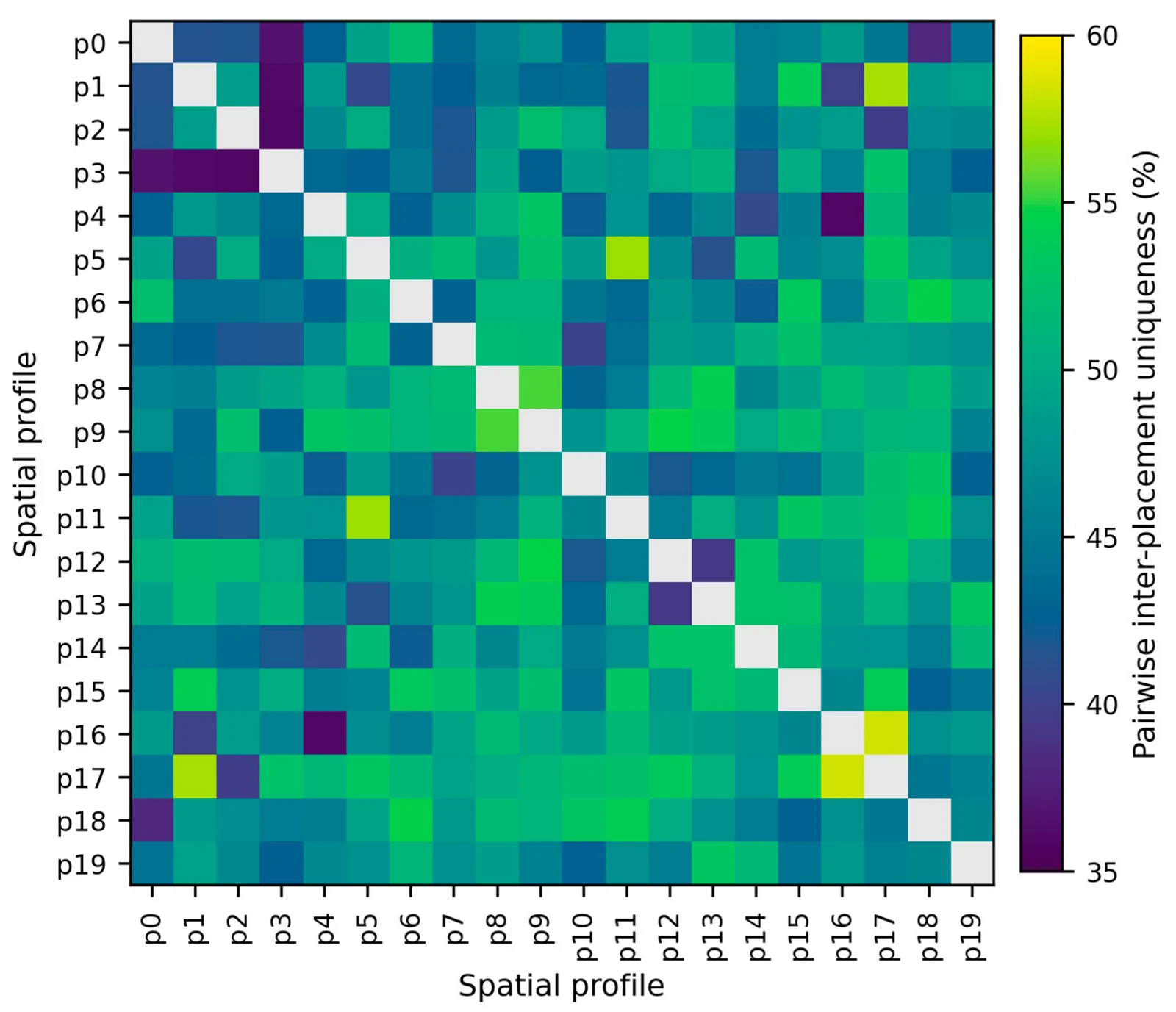
Pairwise inter-placement uniqueness matrix for the twenty spatial SR-APUF profiles. The horizontal and vertical axes correspond to the profile indices (p00-p19). Each off-diagonal cell represents the normalized Hamming distance between the 2048-bit majority-response signatures of two profiles, expressed as a percentage. The color scale encodes the degree of response separation, with values near 50% indicating stronger profile differentiation and lower values indicating increased similarity between profiles. The grey diagonal corresponds to self-comparisons and is not plotted. The minimum observed uniqueness was 35.8887% for profiles p04 and p16, while the maximum was 58.3496% for profiles p16 and p17.

Figure 7 presents the complete pairwise inter-placement uniqueness matrix for the twenty spatial profiles. Across the 190 off-diagonal comparisons, the mean uniqueness was 47.6514%, the median was 47.8760%, and the population standard deviation was 4.3261%. The observed values ranged from 35.8887% for profiles p04 and p16 to 58.3496% for profiles p16 and p17. The mean lies 2.3486 percentage points below the ideal value of 50%, demonstrating substantial placement-dependent response diversity while indicating residual correlation between certain profile pairs. Figure 7 visually illustrates this behavior through the color-coded uniqueness matrix. Most profile pairs cluster around the ideal 50% region, indicating substantial response separation between independently placed implementations. A smaller number of profile pairs exhibit lower uniqueness values, represented by darker regions in the matrix, indicating residual correlation despite physical relocation. The absence of large low-distance regions demonstrates that spatial relocation generally produces distinguishable response personalities across the evaluated profile library.

The observed minimum value of 35.8887% indicates that some profile pairs remain more strongly correlated than others despite their different physical placements. Nevertheless, the value remains substantially above zero, demonstrating that spatial relocation still modifies the challenge–response mapping. Values closer to the ideal 50% indicate greater separation between profiles, whereas lower values indicate increased similarity and reduced inter-placement diversity.

### 6.3. Uniformity

**Figure 8:**
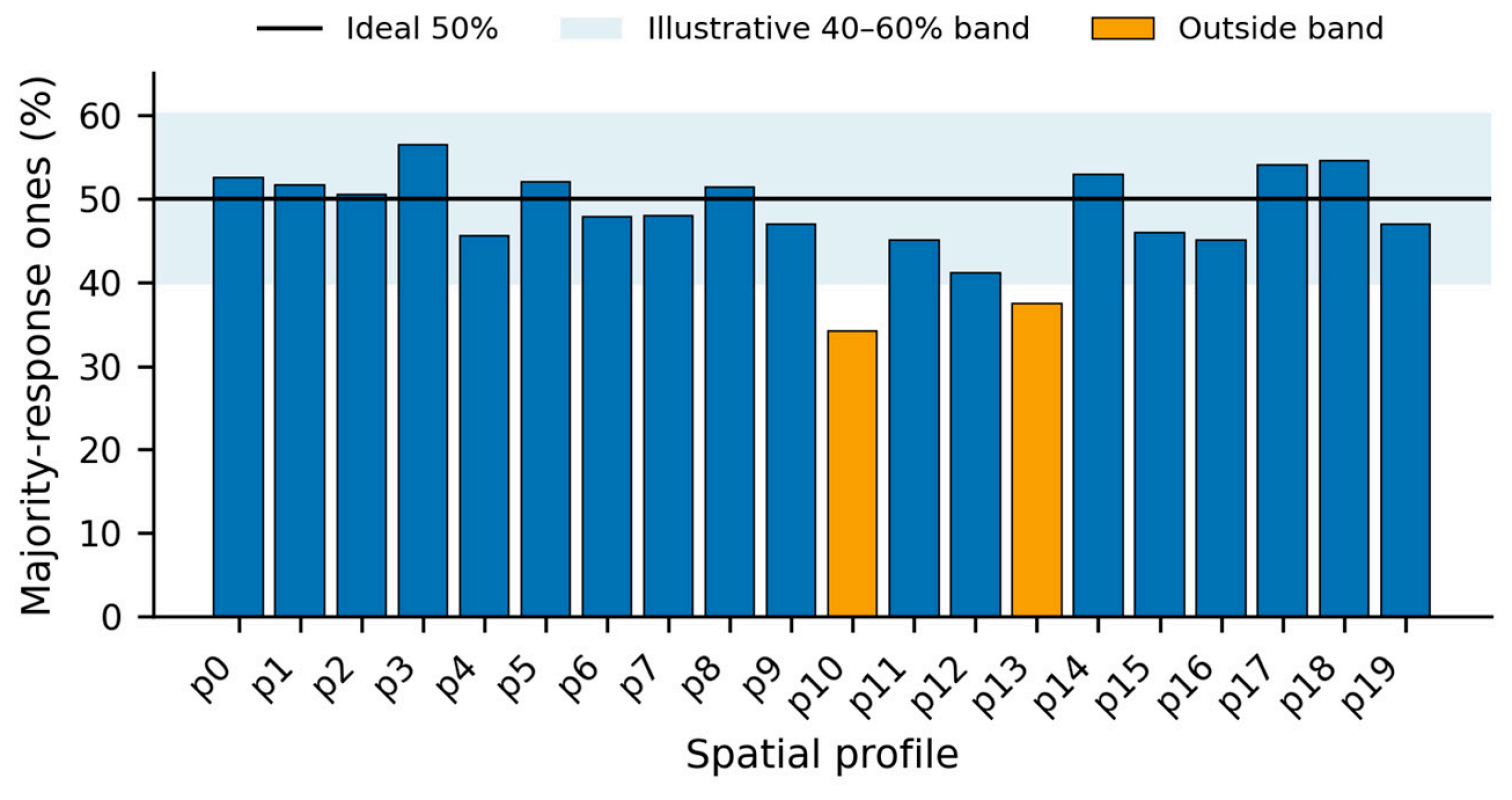
Majority-response uniformity by spatial profile. The shaded band marks the illustrative 40–60% screening interval, and bars outside it are highlighted.

**Figure 9:**
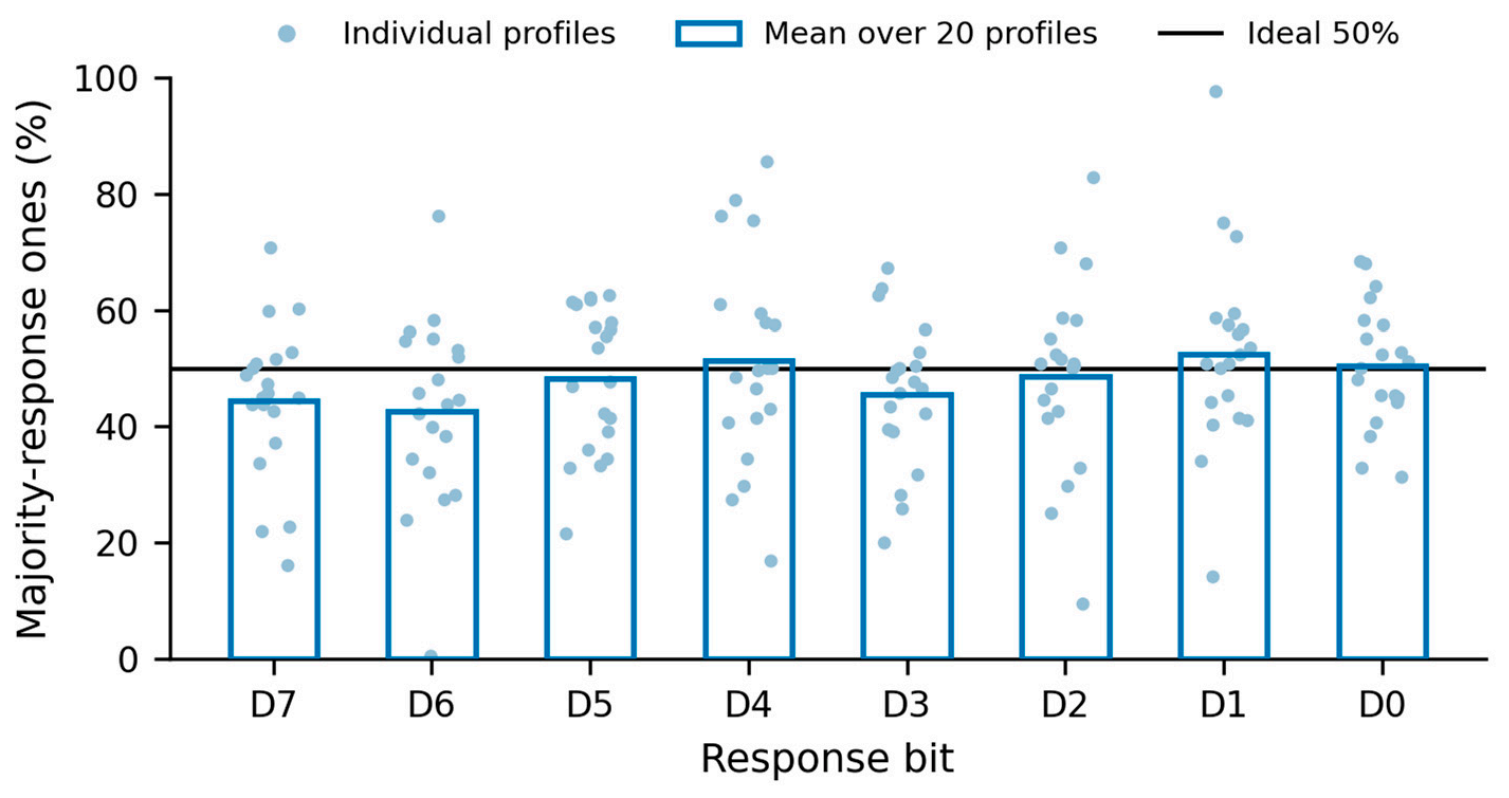
Mean majority-response uniformity of D7 through D0 across twenty profiles. Bars show the mean over the twenty profiles, dots show the individual profiles, and the line marks the ideal 50%.

Uniformity, as presented in Section 2.3.1, is a fundamental PUF quality metric because it measures the balance between logic-0 and logic-1 responses. An ideal PUF produces 0s and 1s with approximately equal probability, corresponding to a uniformity value close to 50%. Significant deviation from 50% indicates response bias, which may originate from systematic delay asymmetries, routing effects, arbiter bias, or implementation-dependent physical characteristics. Excessive bias reduces effective response diversity and may limit the usefulness of the generated responses for authentication or cryptographic applications. Consequently, profile-level uniformity is presented in Table 5 and Figure 8, and per-bit uniformity in Figure 9, and evaluated to identify placement profiles and response positions that exhibit systematic preference toward one logical state.

As shown in Table 5 and Figure 8, mean profile uniformity was 47.9858% (range 34.0820% to 56.4453%). Uniformity quantifies the balance between logic-0 and logic-1 responses. Values near 50% indicate balanced responses, whereas larger deviations indicate response bias and possible implementation-dependent asymmetries. In the context of the illustrative screening criteria discussed in Section 7.3, profiles with uniformity within the range of 40–60% would be considered acceptable candidates for enrollment, while profiles outside this interval may warrant additional investigation depending on application requirements. Profile 10 was below the illustrative 40% lower screening threshold at 34.0820%; profile 13 was also below that threshold at 37.4512%. Per-bit inspection additionally revealed extreme chain-level biases that are hidden by aggregate uniformity (Section 6.4 and Figure 9).

### 6.4. Per-Bit Behavior

Across all profiles, mean bit aliasing was 47.9858% and the median was 50.0%. A total of 120 of the 2048 challenge/response-bit positions exhibited logic-1 frequencies at or below 20% or at or above 80% across the twenty profiles. These positions therefore represent strongly biased response locations and indicate that certain challenge/bit combinations remain dominated by deterministic placement-dependent effects. Particularly strong within-profile bias included D6 of profile 02 at 0.3906% ones and D1 of profile 03 at 97.6563% ones (Figure 9). These observations support response-bit and challenge screening during enrollment.

The profile-level and bit-level biases observed in Figure 8 and Figure 9 indicate that not all spatial implementations contribute equally to response quality. In a practical deployment, enrollment procedures could incorporate profile qualification, bit reliability analysis, or both. The retained response width would then depend on the selected acceptance criteria and the application requirements.

The present study deliberately retains all measured profiles and response bits in order to characterize the complete range of placement-dependent behavior. In particular, profiles p10 and p13 were intentionally retained despite their lower uniformity values to illustrate the full spectrum of placement-dependent responses observed across the measurement campaign. Consequently, the reported results should be interpreted as describing the available response space rather than the amount of cryptographic entropy that could be extracted.

Quantification of extractable key material would require additional analysis of reliability, inter-profile correlation, environmental variation, and the performance of subsequent randomness-extraction or fuzzy-extractor mechanisms, which are beyond the scope of the present work.

### 6.5. Response Diversity, Correlation, and Challenge Sensitivity

The 256 challenges produced between 55 and 104 distinct majority-response bytes per profile, with a mean of 80.4. Empirical byte-distribution statistics therefore show substantial challenge-dependent diversity, although these values must not be interpreted as cryptographic entropy estimates. Response-bit dependence remained visible: several profiles exhibited strong off-diagonal phi correlations, including maximum absolute values above 0.9. The phi coefficient is the Pearson correlation coefficient between two binary variables; it was computed for every pair of the eight response-bit positions across the 256 majority-response challenges of each profile, where values near zero indicate weak dependence and values near +1 or −1 indicate strong positive or negative dependence. Such values indicate that certain response-bit positions do not behave independently and may be influenced by common placement- or routing-dependent effects. One-bit challenge flips generally affected more output bits for higher-indexed challenge positions, indicating non-uniform stage influence rather than an ideal symmetric avalanche response.

### 6.6. Parity Consistency and Data Integrity

The parity equations were consistent for 102,300 of 102,400 captured codewords (99.9023%). Profiles 13 and 15 accounted for the 100 parity-invalid records, with 6 and 94 invalid codewords, respectively. Because the parity bits are generated deterministically from the raw byte, a parity-invalid record indicates an inconsistency between the captured raw byte and the captured parity bits within the same codeword. The parity check is used only as a data-integrity indicator, and no error correction was applied to the reported data. These records cannot presently be attributed to intrinsic PUF instability; they may arise from capture, synchronization, serialization, or communication effects, and they are not treated as independent PUF entropy. All capture files passed record-count and challenge-coverage validation, and no duplicate capture hashes or duplicate majority signatures were found.

### 6.7. Cross-Device Results

All twenty corresponding-profile signatures were nonidentical, and none of the corresponding capture files had equal SHA-256 hashes. The mean corresponding-profile cross-device distance was 2.0703%, the median was 1.9287%, and the population standard deviation across profiles was 0.7341%. The observed range was 0.7324% to 3.5645%, corresponding to 15 to 73 differing bits in a 2048-bit signature (Table 6). Mean raw-bit reliability was 99.8843% on the first board and 99.9104% on the second board.

Across the 380 nonmatching cross-device profile pairs, the mean distance was 47.6537%, the median was 47.9004%, and the observed range was 35.4492% to 58.6914% (Table 7). The distances of all 400 cross-device profile comparisons are provided in the Supplementary Materials. The largest corresponding-profile distance was therefore 31.8847 percentage points below the smallest nonmatching-profile distance. For every profile on the first board, the closest profile on the second board had the same profile index. The complete absence of overlap demonstrates strong preservation of spatial-profile identity across the evaluated devices.

These results should not be interpreted as ideal conventional inter-device uniqueness. Approximately 97.93% of corresponding-profile majority bits match across the tested boards, indicating that deterministic placement and routing dominate the response mapping. The residual device-specific differences are sparse and require stable-bit screening and environmental validation before they can support physical-device key extraction or counterfeit-board rejection. The result is nevertheless strong evidence that each spatial profile forms a reproducible hardware personality that is clearly distinguishable from all nonmatching profiles.

Only two physical devices were evaluated, yielding one independent device pair. Accordingly, 2.0703% characterizes the evaluated VC709 pair and is not reported as a population estimate. Prior statistical work shows that inter-device uniqueness requires multiple physical devices and confidence-interval analysis, while additional challenges and profiles deepen characterization but do not replace independent boards [18].

### 6.8. Profile and Bit Screening Analysis

The aggregate uniformity results indicate that not all spatial profiles contribute equally to the overall response characteristics. In particular, profiles p10 (34.0820%) and p13 (37.4512%) exhibit substantially lower uniformity than the remaining profiles. These profiles were intentionally retained in the reported results to illustrate the full range of placement-dependent behavior observed across the measurement campaign rather than only a pre-selected subset of favorable implementations.

An illustrative screening interval of 40–60% uniformity may be applied in a practical enrollment process to identify profiles exhibiting excessive aggregate bias. The reported statistics were computed without screening, because the objective was to characterize the complete distribution of profile behaviors. Applied post hoc to the measured data, this criterion excludes profiles p10 and p13 and retains 18 of the 20 profiles (90%).

Effect of screening. Excluding p10 and p13 raises the mean profile uniformity from 47.9858% to 49.3435%, whereas the mean raw-bit reliability (99.8840% to 99.8836%) and the mean 8-bit block reliability (99.0820% to 99.0799%) remain practically unchanged, because the two excluded profiles do not differ systematically in reliability from the others. The pair-level and bit-level quantities that follow were computed from the repeat measurement of the first board (see the Data Availability Statement). The mean inter-placement uniqueness of the 153 remaining profile pairs is 47.68%, compared with 47.64% for all 190 pairs, so profile-level screening reduces the number of usable profiles but does not noticeably change inter-placement diversity. At the bit level, applying the same 40–60% interval to each of the eight response bits within a profile retains on average 5.1 of the 8 response bits per retained profile (range 3 to 7), whereas stability screening removes less than 1% of the 2048 challenge/response-bit positions of a profile (99.5% of the positions were perfectly stable over the twenty repetitions). Combining both criteria leaves on average 1288 of the 2048 positions (62.9%; range 761 to 1787) per retained profile, i.e., the usable response width is reduced by about one third in exchange for lower bias and higher stability.

Implications for key extraction. The remaining positions are an upper bound on the available response material, not an estimate of cryptographic entropy. The 2048 positions are generated by eight 8-stage APUFs, each of which is described in the linear additive delay model by only n + 1 = 9 delay parameters [12,13]; the response bits are correlated (absolute phi values above 0.9 in some profiles, Section 6.5), and the challenge space can be fully enumerated. Extracting a 128-bit key, as required for the Ascon-AEAD128 channel of Section 9.7, would therefore require an entropy estimate, helper-data or fuzzy-extractor processing, and privacy amplification, none of which was evaluated here. Profile and bit screening thus improve response quality at the expense of response width, and this reliability-versus-entropy trade-off must be quantified in future key-generation work.

Aggregate profile uniformity alone is insufficient for profile qualification because Figure 9 reveals substantial variation among individual response bits. Certain response positions remain close to the ideal 50% value across most profiles, whereas others exhibit persistent directional bias. Consequently, a practical deployment would likely combine profile-level evaluation with bit-level screening, reliability testing, and enrollment criteria tailored to the requirements of the target authentication system.

The results therefore suggest that profile and bit screening may be useful as a future optimization step rather than a prerequisite for the placement-dependent characterization presented here. Such screening could improve response quality and reproducibility, although potentially at the expense of reducing the available response space. The development and evaluation of profile-selection strategies remain an interesting direction for future work.

## 7. Placement Optimization

### 7.1. Remaining Implementation Degrees of Freedom

The current methodology fixes the location and A6LUT BEL assignment of every switch-stage MUX through profile-specific XDC constraints. Consequently, the physical position of each MUX is explicitly controlled and verified after implementation.

However, several implementation degrees of freedom remain. The current constraints do not explicitly control the relative geometry of the upper and lower MUXes within a switching stage, route symmetry between competing signal paths, or delay balancing across stages. Routing resources were intentionally left unconstrained and were selected automatically by Vivado during implementation. As a result, profile diversity reflects both the prescribed MUX placements and the profile-specific routing realizations generated by Vivado.

A more tightly controlled implementation would additionally constrain local MUX-pair geometry and routing structure, enabling a clearer separation between placement-induced and routing-induced delay variations.

### 7.2. Proposed Paired-MUX Placement

Building upon the current stage-level LOC/BEL placement methodology, the proposed improvement treats each 2 × 2 switch as a matched physical unit. The two multiplexers of each stage are placed in separate LUTs but remain within the same slice or adjacent equivalent slices. The spatial anchor of each of the 64 switch stages is randomized between profiles, while the two MUXes within every stage remain locally matched. This approach is expected to increase profile diversity without introducing excessive deterministic top-versus-bottom path imbalance. This proposed optimization is consistent with prior evidence that FPGA APUF uniqueness depends on placement geometry, physical separation, and structural similarity [20]. Table 8 gives an example of such LOC/BEL assignments for Chain 0, Stage 0 in two profiles.

### 7.3. Candidate-Profile Screening

A prospective enrollment methodology may generate more spatial profiles than are ultimately retained for authentication. Consistent with published PUF quality-evaluation and screening approaches [4,5,15,21,27], profiles exhibiting poor reliability, excessive bias, or limited pairwise diversity could be excluded, after which a subset of qualified profiles could be retained for subsequent enrollment. Response-level screening is also supported by FPGA APUF work in which a bit-self-test mechanism was used to identify and retain reliable response bits [21]. In the present proof of concept, screening is performed offline through repeated measurements, per-bit reliability, majority-response uniformity, and inter-placement distance rather than through an online bit-self-test circuit. Illustrative acceptance criteria are 40–60% uniformity and at least 95% reliability. Any profile screening must be disclosed because it changes the statistical distribution of retained configurations.

## 8. Application to UAV Swarm Sensing Nodes

The present experiments evaluate each spatial APUF profile in a separate full-device configuration. This methodology isolates the placement-dependent behavior of each profile and permits direct analysis of reliability, uniformity, inter-placement uniqueness, and corresponding-profile behavior across two FPGA devices. It does not demonstrate concurrent operation of multiple APUFs, runtime instance selection, response combination, cryptographic key reconstruction, partial reconfiguration, or an end-to-end UAV authentication protocol.

A practical near-term architecture would instantiate twenty or more spatially separated and independently constrained APUFs within one FPGA configuration. A device-specific keyed controller would activate one or more enrolled instances during each authentication epoch, while non-selected instances would remain disabled to limit switching activity, supply disturbance, side-channel leakage and power consumption. Selected instances may be evaluated sequentially to reduce instantaneous current. Their stable response positions would be concatenated, processed using profile-specific helper data, and supplied to a cryptographic key-derivation function. This approach would avoid storing a number of full configuration images of several MB each, which would otherwise require a large storage space, and would eliminate full-device profile-switching latency.

Future implementations should use challenges of at least 64 bits and generate sufficiently wide qualified internal response material through longer APUFs, additional parallel chains, multiple enrolled challenges, selected spatial instances, or a combination of these methods. A nominal response width must not be interpreted directly as entropy. Stable-bit screening, correlation analysis, helper-data processing, entropy assessment, and a domain-separated Key Derivation Function (KDF) are required before 128-bit cryptographic keys can be derived. Raw APUF responses should remain internal to the trusted FPGA security component.

Following validation of the concurrent hardware array, the derived 128-bit material may be integrated with Ascon-AEAD128, standardized for constrained devices in National Institute of Standards and Technology (NIST) SP 800-232 [28]. In the prospective protocol, the UAV identity, nonce, authentication epoch, firmware context, and selected-instance context would be authenticated as associated data, while authentication messages and sensor payloads would be protected by authenticated encryption. The concurrent APUF array, KDF, nonce and epoch management, and Ascon implementation are proposed future work and are not claimed as experimentally demonstrated contributions of this paper.

Required future experiments include post-implementation utilization and power analysis, measured energy and peak-current behavior, sequential and simultaneous activation, inter-instance correlation, environmental reliability, false-reject performance, stable key reconstruction, machine-learning attacks, side-channel analysis, and end-to-end protocol latency and resource utilization.

## 9. Security Discussion and Limitations

### 9.1. Small Challenge and Response Spaces

An 8-bit challenge provides only 256 possible values and can be exhaustively enumerated. The 8-bit raw response also provides limited resolution and must not be used directly as a cryptographic key. The prototype evaluated in this paper is therefore an experimental characterization platform rather than a production strong PUF. Future designs should use substantially longer challenges and wider qualified internal response material, together with controlled access, stable-bit selection, helper-data processing, and a cryptographic KDF.

### 9.2. Machine-Learning Resistance

The current work does not demonstrate immunity or resistance to machine-learning attacks. Standard and XOR Arbiter PUFs have been modeled using logistic regression, evolution strategies, silicon-derived CRPs, reliability information, and chosen correlated challenges [12,13,29,30]. Spatial placement does not change the learnable delay structure of a conventional APUF. Future work must include countermeasures against same-instance prediction, cross-instance transfer, mixed-instance learning, reliability-aware attacks, unknown-instance classification, and attacks with and without knowledge of the active instance.

The objective of the present study is to characterize placement-dependent response diversity rather than to demonstrate resistance against machine-learning attacks. Consequently, the reported results should not be interpreted as evidence that profile switching, profile selection, or profile diversity inherently provides protection against conventional APUF modeling attacks. Demonstration of such protection would require dedicated evaluation using established attack methodologies, including profile-specific, cross-profile, and mixed-profile learning experiments. Such investigations remain outside the scope of the present work and are left for future research.

### 9.3. Environmental Testing

The reported reliability applies only to the evaluated nominal laboratory conditions. Temperature, supply voltage, routing asymmetry, metastability, self-heating, background activity, challenge selection, power cycling, and aging may affect repeatability [4,5,14,15]. Controlled environmental tests and repeated power cycles are required before deployment claims or stable cryptographic key reconstruction can be made.

### 9.4. Exploratory Cross-Device Investigation

The principal experimental objective of this work is to evaluate the reliability, uniformity, and inter-placement diversity of spatially constrained APUF profiles. The second VC709 board was used only as a supplementary exploratory investigation to determine whether corresponding constrained implementations exhibit similar or different response behavior on another physical FPGA. This comparison was not designed as a population-level inter-device uniqueness study, a counterfeit-device rejection experiment, or a statistical characterization of the VC709 device family. Accordingly, the reported corresponding-profile distances describe only the two evaluated boards and are used to support interpretation of the relative contributions of spatial placement and device-dependent manufacturing variation. A broader inter-device study involving additional physical FPGAs, controlled environmental conditions, repeated power cycles, and confidence-interval analysis remains outside the scope of the present proof of concept.

The observed corresponding-profile distance of 2.0703%, compared with a mean non-matching profile distance of 47.6537%, indicates that constrained placement, together with the resulting implementation-generated routing, contributes substantially to the resulting response behavior. Within the limited two-board dataset, corresponding profiles remained clearly distinguishable from non-matching profiles. In the context of the present work, this result is significant because it demonstrates that placement-constrained response profiles can be reproduced across multiple FPGA devices. Such repeatability may be advantageous in prospective swarm-oriented deployments, where qualified placement profiles can be characterized once and subsequently deployed across multiple nodes. However, the present study was not designed as a population-level authentication experiment and does not establish resistance against all possible cloning or impersonation attacks. Only two FPGA devices were available for evaluation, preventing statistically meaningful estimation of false-acceptance rates, false-rejection rates, or authentication thresholds across a larger device population. Consequently, the reported results should be interpreted as evidence of placement-dependent profile behavior and cross-device repeatability rather than a complete security evaluation of an authentication system. Comprehensive analysis of attacker models, authentication thresholds, false acceptance rate (FAR) and false rejection rate (FRR) characteristics, and larger multi-device populations remains future work.

The reported statistics should be interpreted with appropriate caution. The 190 inter-placement distances were derived from twenty spatial profiles and therefore do not constitute 190 independent experimental samples. Similarly, the 380 nonmatching cross-device comparisons originate from only two physical FPGA devices and should not be interpreted as population-level inter-device statistics. In addition, the majority-response reference used for the reliability calculations was derived from the same repeated measurements used for evaluation. Consequently, the reported reliability, uniformity, and distance statistics should be regarded as descriptive measures of the evaluated dataset rather than statistically independent estimates of deployment-level performance. Confidence-interval analysis, bootstrap-based uncertainty estimation, independent enrollment/testing procedures, and larger device populations remain important directions for future work.

Physical unclonability was not evaluated as a primary objective of the present study. Although the supplementary two-board investigation provides insight into the relative influence of placement-dependent and device-dependent effects, the available dataset is insufficient to establish population-level unclonability or counterfeit-device rejection capability. Accordingly, the reported cross-device results should be interpreted as exploratory observations rather than as proof of physical unclonability. Demonstration of unclonability would require evaluation across a substantially larger population of FPGA devices together with appropriate authentication and clone-rejection analyses.

### 9.5. Separate Configurations: Resource Requirements and Storage

The present study evaluates placement-dependent APUF behavior by generating independent full-device FPGA configurations, each corresponding to a distinct spatial implementation profile. On the VC709 platform, each bitstream occupies approximately 29 MB. Consequently, storing twenty qualified profiles requires approximately 580 MB of non-volatile memory. This storage requirement exceeds the 128 MB linear byte-peripheral-interface (BPI) flash available on the VC709 and would necessitate external storage or on-demand bitstream transfer. Table 9 compares the two approaches.

The multi-bitstream approach also incurs configuration latency. Before a particular profile can be evaluated, the device must load the corresponding configuration image, temporarily interrupting the operation of the remaining FPGA design. As an illustration, loading one 29 MB bitstream takes about 8 to 15 s over Joint Test Action Group (JTAG) at an assumed clock of 30 to 15 MHz (one bit per clock cycle) and less than 1 s over any parallel configuration interface that sustains more than 29 MB/s; the actual time depends on the configuration mode and interface used. In terms of resources, the APUF core of the present prototype (core_i in Figure 2) uses 149 LUTs, so twenty concurrent instances would require roughly 3000 LUTs, about 0.7% of the 433,200 LUTs of the XC7VX690T, excluding the selection and response-packing logic. Dynamic power was not measured in this study; it is expected to scale with the number of simultaneously active instances, which is why the unselected instances remain idle.

The proposed concurrent architecture removes the storage and reconfiguration overhead by instantiating multiple qualified APUFs within a single FPGA design and selecting the participating instances at runtime through a verifier-issued selection vector. Unselected APUFs remain idle and do not receive new challenges, thereby reducing unnecessary switching activity.

### 9.6. Concurrent Multi-APUF Limitations

Although the architecture proposed in Section 2.5 addresses the storage and reconfiguration limitations of the multi-bitstream approach, the current measurements cannot be assumed to transfer unchanged to a concurrently instantiated implementation.

Placing multiple APUFs within the same FPGA design may introduce routing congestion, common challenge-distribution fan-out, launch skew, shared supply noise, local heating effects, synthesis and placement optimization interactions, electromagnetic coupling, and information leakage associated with the runtime-selection mechanism.

Furthermore, the response quality of concurrently instantiated APUFs must be re-evaluated. Reliability, uniqueness, inter-profile correlation, environmental stability, and bias may differ from those measured in isolated configurations.

Consequently, the concurrent multi-APUF architecture should currently be regarded as a promising extension motivated by the placement-dependent behavior reported here rather than an experimentally validated replacement for the separate-configuration methodology.

### 9.7. Future Ascon Integration

In the future we intend to leverage the Ascon-AEAD128 protocol [28]. Ascon-AEAD128 uses 128-bit keys and nonces and provides authenticated encryption for constrained devices. The present 8-bit raw responses are insufficient for direct use as Ascon keys.

The Hamming(12,8) parity bits used in the present prototype should not be interpreted as a key-generation or error-correction mechanism. A future authenticated architecture would require dedicated error-correction, helper-data, entropy-conditioning, fuzzy-extractor, and key-derivation mechanisms in addition to the placement-dependent response generation demonstrated here.

A future authenticated system must first demonstrate reproducible derivation of qualified 128-bit key material from wider and independently characterized internal responses. It must then evaluate nonce uniqueness, key separation, associated-data encoding, authentication failure handling, resource utilization, energy, latency, and implementation security. Ascon protocol implementation or performance results fall outside the scope of this work.

### 9.8. Scope and Contribution of the Proof of Concept

Prior studies have established that FPGA placement and routing influence Arbiter-PUF reliability, bias, and uniqueness [4,14,15,18,19,20,21]. Reconfigurable APUF architectures have also been proposed using programmable delay lines and configurable 4 × 4 switch blocks [14,15,16,17], while response-screening mechanisms have been used to improve the quality of FPGA APUF outputs [21]. The contribution of the present proof of concept is therefore not the general observation that APUF behavior depends on physical implementation. Instead, this work provides a systematic profile-level characterization of twenty separately implemented, fine-grained placements of an unchanged eight-chain, eight-stage APUF architecture. Every profile was evaluated exhaustively over all 256 challenges using twenty repeated measurements, and the constrained placement of all 128 switch-stage MUX LUTs was verified through post-implementation LOC and BEL reports. Unlike approaches that modify the switching network, arbiter operating mode, or online response-selection mechanism [16,17,19,21], the evaluated profiles preserve the same logical architecture and differ in their prescribed LUT placement and the resulting Vivado-generated routing. Reference [20] investigates broad geometric placement strategies, whereas the present study evaluates a library of twenty profile-specific LOC/BEL mappings and combines exhaustive challenge evaluation with repeated reliability measurements and a supplementary corresponding-profile comparison across two physical FPGA devices.

The study further distinguishes two forms of variation. On the first FPGA, the twenty placement profiles produced a mean inter-placement distance of 47.6514%, demonstrating that spatial relocation substantially changes the response mapping. In the supplementary two-board investigation, corresponding constrained profiles differed by only 2.0703% on average, whereas nonmatching cross-device profiles differed by 47.6537%. For every profile on the first board, the closest response signature on the second board had the same profile index. Within the limited two-board dataset, these results reveal a hierarchical behavior in which constrained placement and the resulting routing define the dominant response personality and physical-device variation contributes a smaller residual.

The present measurements do not demonstrate concurrent multi-APUF operation, runtime instance selection, cryptographic key reconstruction, or an implemented UAV authentication protocol. They establish an experimental basis for future work in which multiple independently constrained APUFs are instantiated concurrently on one FPGA and qualified internal responses are processed for cryptographic use.

Compared with prior FPGA APUF studies, the emphasis of the present work is not on introducing a new APUF architecture, a new routing methodology, a machine-learning countermeasure, or a key-generation mechanism. Instead, the primary contribution is a systematic profile-level characterization of twenty independently constrained LOC/BEL placement realizations of an otherwise unchanged APUF design. The study combines exhaustive challenge evaluation, repeated measurements, placement verification, and a supplementary two-device comparison to investigate the influence of fine-grained physical implementation on response behavior. Accordingly, the results should be viewed as a placement-diversity characterization study rather than as a complete evaluation of authentication security, machine-learning resistance, or deployment-level PUF performance.

## 10. Conclusions

This paper presented a proof-of-concept evaluation of spatially placed Arbiter PUF profiles for future UAV swarm sensing nodes. Twenty fine-grained spatial profiles of an eight-chain, eight-stage APUF were implemented separately, with one profile in each full-device configuration, and evaluated on two physical Xilinx VC709 boards. On the first device, the prototype achieved 99.8843% mean raw-bit reliability, 99.0820% mean 8-bit block reliability, 47.9858% mean majority-response uniformity, and 47.6514% mean inter-placement uniqueness. These results show that controlled relocation of unchanged logical APUF circuitry can produce stable and substantially distinguishable placement-dependent response mappings.

Across the two boards, corresponding spatial profiles differed by 2.0703% on average, whereas nonmatching cross-device profile pairs differed by 47.6537% on average. The largest corresponding-profile distance was 3.5645%, and the smallest nonmatching-profile distance was 35.4492%, yielding an empirical separation of 31.8847 percentage points in the observed dataset. Deterministic placement and routing therefore define the dominant profile personality, while device-specific manufacturing variation contributes a smaller residual. Because only two boards and one independent device pair were evaluated, the cross-device result is not generalized to the VC709 population and does not establish conventional population-level inter-device uniqueness.

The present proof of concept does not implement the twenty APUFs concurrently, select an active subset at runtime, combine multiple responses, derive cryptographic keys, or evaluate an Ascon-protected authentication protocol. Instead, the results establish the placement-defined reliability and diversity needed to motivate that next stage. A practical future architecture will place twenty or more qualified APUFs concurrently in one FPGA configuration, keep inactive instances electrically quiet, and activate one or a small subset through authenticated device-specific control. Selected instances may be evaluated sequentially, and qualified stable responses will be concatenated and processed through helper data and a cryptographic KDF.

Future APUFs should use challenge widths of at least 64 bits and provide sufficiently wide qualified internal response material for reproducible 128-bit key derivation. Following experimental validation of the concurrent array, the derived keys may be integrated with Ascon-AEAD128 for authentication and protected sensor-data transport. This integration requires new measurements of routing interaction, inter-instance correlation, environmental reliability, power and peak current, side-channel leakage, modeling resistance, key-reconstruction success, nonce management, resource utilization, energy, and protocol latency.

The principal conclusion is therefore limited but experimentally supported: properly constrained spatial placement, together with the resulting implementation-generated routing, can create reliable and distinguishable APUF personalities on FPGA. Concurrent multi-APUF operation and lightweight authenticated-encryption integration constitute the immediate future work enabled by this result.

## Figures and Tables

**Table 1:** Characteristics of the evaluation board.

Parameter	Value
FPGA	XC7VX690T-2FFG1761
Evaluation board	Xilinx VC709
Design tool	Vivado 2021.1
System clock	200 MHz differential
Challenge length	8 bits
Parallel APUF chains	8
Stages per chain	8
Raw response length	8 bits
Hamming parity bits	4
Spatial profiles	20
Repeated measurements per profile	20

**Table 2:** Primitive utilization of the placed design (Vivado 2021.1, report_utilization; one implemented profile, different from the one shown in Figure 2, so the totals differ slightly). LUTn: n-input lookup table; FDRE and FDSE: D flip-flops with synchronous reset and set, respectively; CARRY4: four-bit carry chain; IBUF, IBUFDS, and OBUF: input, differential input, and output buffers; BUFG: global clock buffer.

Primitive	Used in the Placed Design
LUT3	162
FDRE	156
LUT6	52
LUT2	44
LUT4	26
LUT5	22
CARRY4	12
LUT1	11
OBUF	10
IBUF	2
IBUFDS	1
FDSE	1
BUFG	1

**Table 3:** Reliability measured for each spatial profile.

Profile	Raw-Bit Reliability (%)	Block Reliability (%)	Perfectly Stable Challenges (%)	Parity Valid (%)
p0	99.9121	99.2969	95.70	100.0000
p1	99.8340	98.6719	93.75	100.0000
p2	99.8364	98.7305	94.92	100.0000
p3	99.7729	98.2422	94.14	100.0000
p4	99.8999	99.1992	96.88	100.0000
p5	99.8291	98.6328	94.92	100.0000
p6	99.8828	99.0625	95.70	100.0000
p7	99.8633	98.9648	94.92	100.0000
p8	99.9414	99.5312	96.88	100.0000
p9	99.9219	99.3750	96.48	100.0000
p10	99.9146	99.3164	96.48	100.0000
p11	99.8853	99.0820	94.92	100.0000
p12	99.9463	99.5703	98.05	100.0000
p13	99.8608	98.8867	95.31	99.8828
p14	99.8877	99.1016	96.09	100.0000
p15	99.8364	98.6914	95.70	98.1641
p16	99.9365	99.4922	97.66	100.0000
p17	99.9023	99.2188	96.09	100.0000
p18	99.9365	99.4922	98.05	100.0000
p19	99.8804	99.0820	96.09	100.0000
Overall	99.8843	99.0820	—	99.9023

**Table 4:** Summary statistics of the pairwise inter-placement uniqueness.

Statistic	Value
Spatial profiles	20
Profile pairs	190
Mean inter-placement uniqueness	47.6514%
Minimum	35.8887%
Maximum	58.3496%
Median	47.8760%
Standard deviation	4.3261% (population)

**Table 5:** Profile uniformity.

Profile	Response Ones (%)
p0	52.5391
p1	51.6113
p2	50.4883
p3	56.4453
p4	45.5078
p5	52.0508
p6	47.8027
p7	47.9492
p8	51.4160
p9	46.9238
p10	34.0820
p11	44.9707
p12	41.0645
p13	37.4512
p14	52.9297
p15	45.9473
p16	45.0684
p17	54.0039
p18	54.5898
p19	46.8750
**Overall mean**	**47.9858**

**Table 6:** Corresponding-profile cross-device comparison summary.

Metric	Value
Physical FPGA devices	2
Corresponding profile comparisons	20
Bits per majority signature	2048
Mean corresponding-profile distance	2.0703%
Median	1.9287%
Minimum	0.7324% (p17; 15 bits)
Maximum	3.5645% (p03; 73 bits)
Population SD across profiles	0.7341%
Mean reliability, first device	99.8843%
Mean reliability, second device	99.9104%
Duplicate corresponding capture hashes	0

**Table 7:** Matching- and nonmatching-profile cross-device regimes.

Comparison Class	Count	Mean	Range
Same profile, different FPGA	20	2.0703%	0.7324–3.5645%
Different profile, different FPGA	380	47.6537%	35.4492–58.6914%

**Table 8:** Example LOC/BEL assignments for Chain 0, Stage 0 in two spatial profiles. A6LUT–D6LUT denote the four LUT sites of a slice.

Example Item	Profile A	Profile B
Chain 0, Stage 0—upper MUX	SLICE_X40Y10/A6LUT	SLICE_X117Y83/C6LUT
Chain 0, Stage 0—lower MUX	SLICE_X40Y10/B6LUT	SLICE_X117Y83/D6LUT

**Table 9:** Comparison between the separate-configuration approach evaluated in this study and the proposed concurrent single-bitstream architecture.

Characteristic	Separate Configurations (This Study)	Concurrent Single Bitstream (Proposed)
FPGA configurations	20 full-device bitstreams	Single bitstream
Storage requirement	~580 MB	~29 MB
Runtime profile selection	Requires reconfiguration	Immediate via selection vector
Configuration latency (illustrative)	About 8 to 15 s per profile over JTAG at an assumed 30 to 15 Mb/s; below 1 s over a parallel interface above 29 MB/s	None (selection vector only)
FPGA resource utilization	One APUF core (149 LUTs)	Twenty cores: about 3000 LUTs (0.7% of the device), plus selection logic
Dynamic power	Not measured	Not measured; idle instances add no switching activity
Experimental validation	Demonstrated	Not yet demonstrated

## Data Availability

The original contributions presented in this study are included in the Supplementary Material. The Supplementary Material contains the raw captures, the post-implementation placement reports, the bitstreams, and the pairwise and cross-device analysis files. The captures of the first board in the Supplementary Material are a repeat measurement: the first-board statistics of Section 6.1, Section 6.2, Section 6.3, Section 6.4, Section 6.5 and Section 6.6 were computed from the original captures, whereas the cross-device comparison of Section 6.7, the pair-level and bit-level quantities of Section 6.8, and Figure 6, Figure 7 and Figure 9 use the repeat measurement, whose per-profile reliability, block-reliability, and uniformity values agree with the original ones to within 0.4 percentage points. Further inquiries, including requests for the VHDL source and the constraint files, can be directed to the corresponding author(s).

## Supplementary Materials

The following supporting information can be downloaded at: https://www.mdpi.com/article/10.3390/s26216719/s1, the raw capture files of both boards, the post-implementation placement reports, the bitstreams, and the pairwise and cross-device analysis files.

## Abbreviations

The following abbreviations are used in this manuscript:

AEAD	Authenticated encryption with associated data
APUF	Arbiter physical unclonable function
BEL	Basic element of logic
BER	Bit-error rate
BPI	Byte peripheral interface
CLB	Configurable logic block
CRP	Challenge–response pair
FAR	False acceptance rate
FPGA	Field-programmable gate array
FRR	False rejection rate
HD	Hamming distance
IoD	Internet of Drones
KDF	Key derivation function
LED	Light-emitting diode
LOC	Physical location (placement constraint)
LUT	Lookup table
MUX	Multiplexer
NIST	National Institute of Standards and Technology
PUF	Physical unclonable function
SD	Standard deviation
SHA	Secure Hash Algorithm
SLICEL	Logic slice
SLICEM	Logic-and-memory slice
SR-APUF	Spatially reconfigurable arbiter PUF
UART	Universal asynchronous receiver–transmitter
UAV	Unmanned aerial vehicle
VHDL	VHSIC hardware description language
XDC	Xilinx Design Constraints
XOR	Exclusive OR
JTAG	Joint Test Action Group
